# Effect of pomegranate peel powder‐infused multigrain chapatti on diabetes prevention: A randomized clinical trial

**DOI:** 10.1002/fsn3.4134

**Published:** 2024-04-12

**Authors:** Muhammad Zubair, Aftab Ahmed, Muhammad Afzaal, Farhan Saeed, Zargham Faisal, Aasma Asghar, Noor Akram, Salim Manoharadas, Asad Nawaz, Degnet Teferi Asres

**Affiliations:** ^1^ Department of Food and Nutrition Government College University Faisalabad Faisalabad Pakistan; ^2^ Department of Nutritional Sciences Government College University Faisalabad Faisalabad Pakistan; ^3^ Department of Food Science Government College University Faisalabad Faisalabad Pakistan; ^4^ Department of Human Nutrition and Dietetics Iqra University Karachi Karchi Pakistan; ^5^ Food Safety & Biotechnology Lab Department of Food Science Government College University Faisalabad Faisalabad Pakistan; ^6^ Department of Botany and Microbiology College of Science, King Saud University Riyadh Saudi Arabia; ^7^ Shenzhen Key Laboratory of Marine Microbiome Engineering Institute for Advanced Study, Shenzhen University Shenzhen China; ^8^ Bahir Dar Food and Nutrition Research Center Bahir Dar Institute of Technology, Bahir Dar University Bahir Dar Ethiopia

**Keywords:** antioxidant, diabetes, multi‐grain, nutrition, pomegranate

## Abstract

Diabetes mellitus is a metabolic and chronic disease linked to lifestyle factors like dietary patterns and physical inactivity. This randomized clinical study aimed to develop a novel dietary intervention using pomegranate peel powder‐based multigrain chapatti to prevent diabetes. The product was formulated by incorporating pomegranate peel powder into a mixture of wheat flour, pearl flour, millet flour, and chickpea flour. The study included the formulation of various treatments (T_c_, T_1_, T_2_, and T_3_) following product development, and these treatments were subjected to comprehensive assessments. The nutritional composition and antioxidant potential of the pomegranate peel powder‐based multigrain chapatti were analyzed. Sensory attributes, including taste, texture, and overall acceptability, were evaluated. Additionally, biochemical analyses, including blood glucose levels and HbA1C, were conducted to assess the impact of the interventions on blood glucose metabolism. The results revealed that the nutritional profile and phytochemical potential of the product improved significantly in treatment T_3_, which contained 15% pomegranate juice. Overall acceptability was found to be high for T_3_, indicating that the inclusion of pomegranate peel powder was well received in terms of taste and sensory qualities. Importantly, the clinical trial demonstrated positive outcomes in the intervention group receiving the pomegranate peel powder‐based multigrain chapatti. Blood glucose analysis and HbA1C assessments indicated that the consumption of this innovative dietary product contributed to improved blood glucose metabolism, suggesting its potential as a preventive strategy for diabetes.

## Graphical abstract

Figure 1 has been presented as a graphical abstract that implies the blood glucose profile of diabetic patients after intervention.

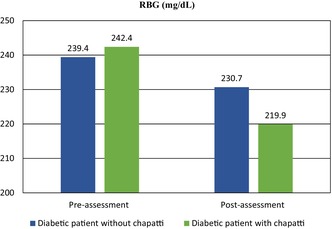

## 1 INTRODUCTION

**FIGURE 1:**
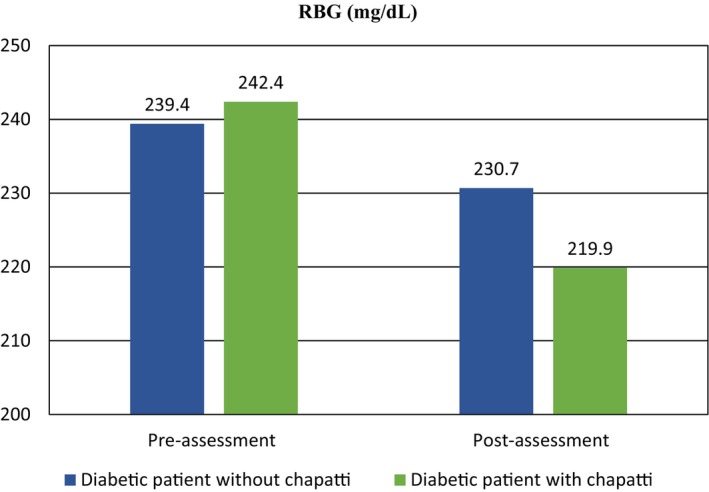
Comparison of random blood glucose levels in the selected groups of study.

Diabetes mellitus is a chronic metabolic disorder characterized by elevated blood glucose levels resulting from impaired insulin action and/or secretion, accompanied by dyslipidemia. It is classified into two types: Type 1 diabetes mellitus, an autoimmune disorder characterized by insulin deficiency, and Type 2 diabetes mellitus, primarily associated with insulin resistance and metabolic dysfunction leading to hyperglycemia, dyslipidemia, and carbohydrate metabolism disorders (Faisal et al., 2022). According to the World Health Organization (WHO), around 422 million people worldwide have diabetes, with an estimated global prevalence of 9.3% in 2019 and projected to reach 10.2% by 2030 and 10.9% by 2045 (Saeedi et al., 2019). Developing nations account for 75% of the diabetic population, with significant increases observed in countries like India and Pakistan (Khan et al., 2023). Accurate estimates and projections are crucial for planning and monitoring diabetes prevention and treatment strategies (Figure 1). Dietary approaches, including plant‐based Mediterranean diets, soy and cod proteins, and low‐fat, plant‐based, and cereal‐based diets, have shown promise in managing diabetes and controlling hyperglycemia (Jardine et al., 2021).

Multigrain value‐added products have gained popularity due to their enhanced nutritional profile, including higher protein digestibility, a lower glycemic index, increased dietary fiber, and greater resistant starch content (Chauhan et al., 2017). Roti, a type of Indian flatbread prepared from multigrain flour combined with pomegranate peel powder (PPP), offers several health benefits, such as a lower glycemic index compared to whole wheat flour and potential risk reduction for non‐communicable diseases like diabetes mellitus (Tomer et al., 2018). Pomegranate (*Punica granatum*), cultivated in the Middle East and Western countries, is recognized for its medicinal properties and abundance of natural antioxidants and nutrients (Kandylis & Kokkinomagoulos, 2020). Pomegranate peel, considered a byproduct after juice extraction, can be utilized in the production of value‐added food products, owing to its rich content of phytochemicals, phenolic compounds, and essential amino acids (Tátraaljai et al., 2022). PPP exhibits various medicinal effects, including antibacterial, antioxidative, immune‐boosting, and wound‐healing properties (Hanafy et al., 2021; Saparbekova et al., 2022). Its incorporation into food products enhances stability, physiological properties, and nutritional benefits. PPP has been successfully integrated into diverse food items, such as jellies, meat products, edible oils, and bakery items (El‐Shamy & Farag, 2021).

Wheat, scientifically known as *Triticum aestivum* L., is a highly nutritious cereal crop consumed by a significant portion of the global population. It is rich in protein, carbohydrates, fats, crude fiber, and various minerals and vitamins, making it an essential dietary source (AbaDura, 2022; Ghafoor et al., 2017). Despite its nutritional value, wheat lacks certain essential amino acids, which can be addressed by incorporating other cereals into wheat‐based products (Martins et al., 2017). Chickpea (*Cicer arietinum* L.), characterized by its carbohydrates, protein, dietary fiber, and micronutrient content, offers functional properties suitable for gluten‐free bakery products (Sofi et al., 2020; Xing et al., 2021). Pearl millet (*Pennisetum glaucum*) is another valuable grain rich in protein, minerals, and dietary fiber. It possesses biochemical properties such as a low glycemic index and higher antioxidant activities, which may contribute to managing conditions like diabetes (Mawouma et al., 2022). The incorporation of pearl millet flour, along with other multigrain flours like chickpea flour, wheat, and pomegranate powder, offers a promising approach to developing bakery products with enhanced nutritional and health benefits (Meenu et al., 2022).

Fast food consumption has become popular, but the refined flour used in its preparation poses health risks. In contrast, multigrain flour products, containing grains like oats, pearl millets, maize, bengal gram, and ragi, offer a favorable nutritional profile. They aid in weight control and reduce the risk of diabetes, heart disease, and bowel cancer (De, 2020). Incorporating multigrain flour with legume grains and plant‐based materials, such as PPP, in bakery products, like bread, can effectively modulate carbohydrate metabolism and lower glucose levels. Studies have explored the availability and properties of composite flour‐based bread (Olagunju, 2019). Diabetes prevalence is high in developing countries with limited access to healthcare. Therefore, cost‐effective, and innovative strategies are needed, such as nutrient‐rich and anti‐diabetic foods, like multigrain flour products, which play a crucial role in diabetes management (Foster et al., 2020). The food industry generates substantial waste, including pomegranate peel, which has high nutritional value. The current study aimed to incorporate PPP into value‐added chapatti, evaluate its nutritional composition, and examine its impact on diabetes.

## 2 MATERIALS AND METHODS

### 2.1 Procurement of raw materials

Wheat (Galaxy‐2013) flour, chickpea (CM‐98) flour, pearl millet (BMR‐2) flour, and pomegranate (Kandhari) peel powder were procured from a local market in Faisalabad, Pakistan. The efficacy study was conducted in the Department of Food Science and Technology, Government College University, Faisalabad, Pakistan.

### 2.2 Preparations of PPP‐based multigrain chapatti

For chapatti preparation, the dough was made by adding 100 g of wheat flour to 140 mL of hot water and kneading until soft. To create different treatments, multigrain flour with chickpea, pearl millet, and PPP was used as a replacement for wheat flour. In the T_1_ treatment, the ratio of chickpea flour, pearl millet flour, and PPP was 25%, 20%, and 5%, respectively. In T_2_ and T_3_ treatments, the ratios were 20% and 15% for chickpea flour, 25% and 30% for pearl millet flour, and 10% and 15% for PPP, respectively. These treatments were compared to a control group (T_C_) without any replacement, as shown in Table 1.

**TABLE 1:** The treatment plan for product development.

	Wheat flour (g)	Pearl millet flour (g)	Chickpea flour (g)	PPP (g)
T_C_	100	0	0	0
T_1_	50	20	25	5
T_2_	45	25	20	10
T_3_	40	30	15	15

### 2.3 Nutritional composition of chapatti

#### 2.3.1 Determination of moisture

The amount of moisture in a sample was measured with a moisture analyzer (Drying Oven 202‐0A) described in AOAC (2000). A 5 g powder sample of multigrain‐based PPP chapatti was put on the metal plate with a round bottom, and the plate was put into the moisture analyzer at 100°C. The sample was dried until its weight stayed the same. After that, it was cooled and weighed. The sample's water content was used to describe how much weight it gained or lost.

$$\text{Moisture}\%=\frac{\text{Sampl}e^{\prime }s\;\text{weight before drying}-\text{Sampl}e^{\prime }s\;\text{weight after drying}}{\text{Weight of sample}}\times 100$$

#### 2.3.2 Determination of protein

The protein content in the sample was measured using Kjeldahl equipment following the methods outlined in AOAC (2016). A digestion mixture was prepared by mixing CuSO_4_, K_2_SO_4_, and FeSO_4_ in a ratio of 5:94:1. 1 g of the sample, along with a few drops of digestion mixture and 25 mL of concentrated sulfuric acid, were placed in a digestion flask and digested until clear. The resulting solution was transferred to a 250 mL volumetric flask. The 50 mL sample from the volumetric flask was mixed with 10 mL of NaOH. To distill the sample solution, 25 mL of boric acid solution was added. Methyl red was used as an indicator, and N/10 H_2_SO_4_ was used for titration. The amount of nitrogen is calculated by the following equation:

$$\text{Nitrogen}\;\left(\%\right)=\frac{0.014\mathrm{ml}\;\text{of}\;0.1N\;H2\mathrm{SO}4\;\text{used}\times \text{volume of diluted sample}}{\text{Weight of sample}\times \text{volume of aliquot}\;}\times 100$$

$$\text{Crude Protein}\;\left(\%\right)=\text{Nitrogen}\;\left(\%\right)\times 6.25$$

#### 2.3.3 Fat determination

The AOAC (2000) method was used to measure the crude fat content in a sample using a Soxhlet apparatus. A 2–3 g portion of a moisture‐free chapatti sample was placed in an extraction thimble. The thimble underwent a 6‐h Soxhlet extraction process with petroleum ether as the solvent for fat extraction. After removing the petroleum ether, the remaining substance was dried in an oven at 80°C. The sample was then transferred to a desiccator to cool to room temperature and weighed. The decrease in thimble weight indicated a reduction in crude fat content, which was expressed as a percentage of fat in the sample.

$$\text{Crude}\;\mathrm{Fat}\;\left(\%\right)=\frac{\text{Initial weight of sample}-\text{Final weight of sample}}{\text{Weight of sample}}$$

#### 2.3.4 Determination of ash

The ash content of the sample was determined using a muffle furnace apparatus following the methodology outlined by Harris and Marshall (2017). A 5 g chapatti sample was placed in a crucible and heated to evaporate any remaining moisture. The sample was then introduced into the muffle furnace, maintained at a temperature of 550°C, until white or gray ash was formed. The ash was subsequently dried in a desiccator to measure its final weight.

$$\mathrm{Ash}\;\left(\%\right)=\frac{100\;\left(\text{weight of crucible with}\;\mathrm{ash}-\text{weight of crucible}\right)}{\left(\text{weight of crucible with sample}-\text{weight of crucible}\right)}$$

#### 2.3.5 Determination of crude fiber

The fiber content of the sample was estimated using the recognized analytical technique, AOAC (2000). A three‐to‐five‐gram chapatti sample was digested with 200 mL of 0.255 N H2SO4 in a conical flask, followed by boiling for 30 min. The digest was thoroughly rinsed with distilled water. A second digestion was performed using 200 mL of 0.313 N NaOH and a reflux condenser for 30 min. The resulting precipitates were filtered through a Gooch crucible and washed with hot distilled water and ethyl alcohol. The dried Gooch crucible was weighed and melted at 550–6000°C in a muffle furnace, and the weight change was recorded to calculate the amount of crude fiber.

$$\text{Crude Fiber}\%=\frac{\mathrm{Dry}\;\text{residue crucible}\left(g\right)–\left(\text{ignited residue}+\text{crucible}\right)}{\;\text{Sample weight}\;\left(g\right)\times \left(100\%-\text{sample moisture}\%\right)}$$

### 2.4 Sensory evaluation

The sensory qualities of color, flavor, taste, texture, and overall acceptability of the multigrain‐based PPP chapatti were assessed following the method by Taiwo and Kehinde (2019). A panel of 10 participants, including both males and females, conducted the sensory evaluation. The evaluation was carried out in four separate sessions, with each panelist randomly receiving a sample. The samples were presented on disposable plates along with a structured questionnaire. Water and unsalted crackers were provided to cleanse the palate between samples. The sensory evaluation utilized a nine‐point hedonic scale, ranging from 1 (severe dislike) to 9 (extreme like), to score the various characteristics of the chapatti sample.

### 2.5 Determination of polyphenol content

The total phenolic content of the samples was determined using the Folin–Ciocalteau method described by Chang et al. (2020). Folin–Ciocalteau reagent was mixed with a saturated sodium carbonate solution and distilled water in a flask. The mixture was incubated at 37°C for 30 min, and the absorbance was measured at 765 nm using a UV‐Vis spectrophotometer. A gallic acid solution‐based standard curve was used to compare the results. The total phenolic content was expressed as milligrams of gallic acid equivalents per gram of fresh weight (mg GAE/g FW). Calculations were performed using specific formulas.

C=c×V/m

where *C* = total phenolic contents, *c* = concentration of gallic acid, *V* = volume of extract, and *m* = weight of sample.

### 2.6 Determination of antioxidant activity

The antioxidant activity of multigrain‐based PPP chapatti was assessed using the DPPH free radical method, following the procedure outlined by Yu et al. (2002). A 1 g sample was refluxed with 10 mL of methanol for 30 min, and the resulting extract was centrifuged at 5000 rpm for 10 min. The supernatant was used for analysis. For each sample, 0.1 mL of freshly prepared DPPH solution was added to 3.9 mL of methanol. The absorbance at 517 nm was recorded for 1 h until the methanol was blanked. The difference in absorbance, with and without antioxidants, determined the radical scavenging capacity, expressed as the percentage of DPPH.

### 2.7 Efficacy plan

#### 2.7.1 Selection of subjects

A total of 20 participants (30–55 years old) with type‐2 diabetes from Faisalabad, Pakistan, including both males and females, were selected for the study. The inclusion criteria of the subjects were age between 30 and 55 years, confirmed diagnosis of type‐2 diabetes, stable health condition without any other chronic disease, and consistent dietary habits for the past 3 months. The exclusion criteria included type‐1 diabetes, known allergies to pomegranate or any other component of multigrain chapatti, pregnant and lactating mothers, and an inability to follow dietary restrictions. The subjects were divided into two groups. The first group received a value‐added chapatti treatment with PPP, while the second group did not receive the treatment. The therapy schedule lasted for 4 weeks, during which the participants were instructed to include 2 chapattis (approximately 4 servings) in their daily meal plan.

#### 2.7.2 Anthropometric assessment

The nutritional status of all participants, including both males and females, was assessed by measuring their body weight (in kilograms), and height (in meters), and calculating their body mass index (BMI) using the protocols described by Eze et al. (2017). The weight of the selected individuals was determined using a weight balance, with participants standing barefoot and wearing minimal clothing. The height of participants was measured using a non‐stretchable measuring tape. Subsequently, the average weight and height of the participants were calculated. The BMI of the individuals was measured by the following equation:

$$\mathrm{BMI}=\frac{\text{Weight in}\;\mathrm{kg}}{\text{Height in meter square}}$$

### 2.8 Biochemical analysis

#### 2.8.1 Random blood glucose (RBG) and fasting blood glucose (FBG)

The RBG GOD‐POD method described by Ahn et al. (2011) was used for analysis. Both RBG and FBG were analyzed using this method. RBG blood samples were collected randomly, while FBG samples were collected in a fasting state. Blood samples were collected in labeled test tubes: blank, standard, and test. A glucose color reagent (1000 μL) was added to each test tube. The blank tube received distilled water (10 μL), the standard tube received a standard solution (10 μL), and the test tube received plasma (10 μL). The reagents were mixed and incubated at 37°C for 15 min. Optical density (OD) was measured at 530 nm for the blank, standard, and test‐labeled tubes, resulting in ratios of 0.02, 0.45, and 0.58, respectively. The following formula was used to calculate the glucose concentration:

$$\begin{array}{c} \text{Concentration of glucose}\\ =\frac{\mathrm{OD}\;\text{of test}-\mathrm{OD}\;\text{of blank}}{\mathrm{OD}\;\text{of standard}-\mathrm{OD}\;\text{of blank}}\times \text{concentration of standard} \end{array}$$

#### 2.8.2 HbA1C

HbA1c levels were determined using the Tosoh hemoglobin A1c method with HPLC apparatus (Gong et al., 2016). Blood samples were collected and diluted with hemolyzing buffer. The diluted blood was then passed through an ion exchange column with pre‐filtering. Hemoglobin was eluted using three buffers, and dual‐wavelength detection was utilized to measure the peak. Two‐point calibrators were employed for quantification. The elution time for each blood sample was 3 min, and unstable A1c was retained as a separate peak. Barcodes were used, and the results included peak IDs, %Hb, retention times, sample ID, peak areas, and a full chromatogram.

### 2.9 Statistical analysis

The data was analyzed using SPSS version 2022 software to determine the significance level. A one‐way ANOVA and *t*‐test were used for the intervention study. Mean values, percentages, and graphical representations were employed for data visualization (Everitt et al., 2022).

## 3 RESULTS AND DISCUSSION

### 3.1 Proximate analysis

#### 3.1.1 Moisture

**FIGURE 2:**
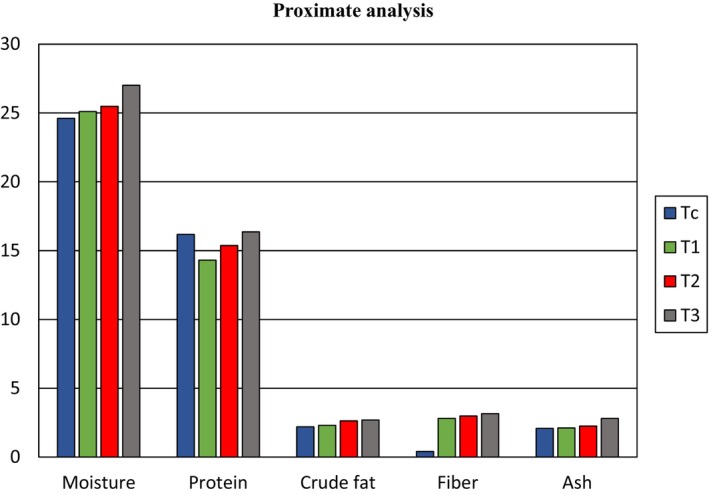
Mean proximate analysis values for chapattis across all treatment groups.

All proximate analyses are shown in Figure 2. Moisture content is a critical factor influencing the physical attributes and shelf life of food products, as well as their quality, freshness, and microbial resistance (Pinela & Ferreira, 2017). It plays a significant role in determining the product's characteristics and longevity. In the case of multigrain PPP chapatti, Table 2 displays the results of moisture content. Significant differences were observed among all treatments and the control group, with moisture content increasing from Tc (24.61 ± 0.03) to T_3_ (27.01 ± 0.69). The lowest moisture value was found in T_c_ with 5% PPP, while the highest was in T_3_ with 15% PPP. These findings align with Pamisetty et al. (2020), who studied the effects of punicic acid‐enriched bread on physicochemical properties, noting an increase in moisture content. A similar trend in moisture content was observed in multigrain chapatti enriched with PPP. Tharshini (2018) also reported higher moisture content in a product made with wheat flour, soybean flour, and PPP, while moisture content increased with higher PPP amounts. Consequently, the moisture content observed in value‐added chapatti aligns with these previous investigations. Additionally, Paul and Bhattacharyya (2015) found higher moisture content in cookies prepared with the addition of PPP, consistent with the current results.

**TABLE 2:** The mean value of proximate analysis of all treatment chapattis.

	T_C_	T_1_	T_2_	T_3_
Moisture	24.61 ± 0.03	25.10 ± 0.18	25.48 ± 0.21	27.01 ± 0.69
Fat	2.20 ± 0.01	2.31 ± 0.19	2.63 ± 0.09	2.69 ± 0.05
Fiber	0.41 ± 0.01	2.81 ± 0.05	2.98 ± 0.02	3.16 ± 0.01
Protein	16.18 ± 0.08	14.31 ± 0.14	15.37 ± 0.05	16.36 ± 0.86
Ash	2.08 ± 0.00	2.12 ± 0.02	2.25 ± 0.02	2.81 ± 0.02

#### 3.1.2 Crude protein

Protein determination is a crucial aspect of proximate analysis. Table 2 presents the protein analysis of value‐added chapatti. Significant variations (*p* < .05) in protein content were observed among the different treatments and the control group. The highest protein content was found in T_3_ (16.36 ± 0.86) with increased PPP concentration, while the lowest value was observed in T_1_ (14.31 ± 0.14). Tharshini (2018) reported higher protein content in cakes prepared with wheat flour, soybean flour, and PPP, consistent with our study on value‐added chapatti. Paul and Bhattacharyya (2015) found that value‐added products incorporating PPP exhibited higher protein content with increased PPP amounts, which aligns with our results. Similarly, Pamisetty et al. (2020) observed increased protein content in multigrain chapatti enriched with PPP, indicating a similar trend.

#### 3.1.3 Ash content

The term “ash content” denotes the inorganic residue that remains following the process of either combustion or complete oxidation of organic matter within a food product. The determination of the ash level of a food is a crucial component of proximate analysis, which is conducted to evaluate its nutritional value. This analysis also considers the ash content as a significant quality characteristic for certain food ingredients. The analysis aids in the determination of the quantity and composition of minerals present in food, a crucial factor, as the mineral content can influence the physiochemical characteristics of food products and inhibit the proliferation of microbes. In the case of multigrain PPP chapatti treatments, significant variations in ash content were observed compared to the control group (T_c_ to T_3_). Table 2 highlights the results, demonstrating a significant (*p* < .05) increase in ash content from T_c_ (2.08 ± 0.00) to T_3_ (2.81 ± 0.02). The lowest ash content was found in T_c_ (2.08 ± 0.00), while T_3_ (2.81 ± 0.02) exhibited the highest value with 15% PPP. This alteration in ash content reflects the mineral trends within the treatments. Mehder (2017) observed higher ash content in PPP‐fortified pan bread as PPP amounts increased, consistent with our findings, the higher ash content in spaghetti incorporated with PPP, aligning with our study on value‐added chapatti. Similarly, Topkaya and Isik (2019) found increased ash content in muffin cake formulated with PPP as PPP amounts increased, reflecting a similar trend observed in value‐added chapatti.

#### 3.1.4 Crude fat

Fat is a crucial component in proximate analysis due to its role as a primary energy source and its contribution to various metabolic functions in the body. The analysis of treatment variances revealed a significant (*p* < .05) change in fat content. Table 2 displays the results of crude fat in different multigrain PPP chapatti treatments: T_1_ (2.31 ± 0.19), T_2_ (2.63 ± 0.09), and T_3_ (2.69 ± 0.05), with increasing PPP concentration, as well as the control group T_c_ (2.20 ± 0.01) without PPP. The highest and lowest crude fat values were observed in T_3_ (2.69 ± 0.05) and T_c_ (2.20 ± 0.01), respectively. The observed elevation in fat content resulting from the addition of PPP can be attributed to the comparatively higher lipid content of PPP in comparison to wheat flour. Additionally, it is plausible that the fat extraction process may be influenced by potential interactions between PPP and other ingredients. Bourekoua et al. (2018) found an increase in fat content when PPP was used as a supplement in pan bread, which aligns with our findings. Ismail et al. (2014) reported similar trends in free fatty acid levels in control cookies and PPP‐added cookies, corroborating our observations on fat content in value‐added chapatti. Tharshini (2018) discovered higher fat content in cakes prepared with wheat flour, soybean flour, and PPP compared to the control cake, consistent with the results of our study that fat content increased with higher PPP amounts. Thus, our investigation demonstrates a comparable trend in fat content for value‐added chapatti, aligning with previous research.

#### 3.1.5 Crude fiber

Fiber, a complex carbohydrate that enhances intestinal digestion, is an important component in proximate analysis. Table 2 presents significant (*p* < .05) results of crude fiber in different multigrain PPP chapatti treatments and the control. Fiber content increased with higher PPP concentrations, ranging from T_c_ (0.41 ± 0.01) to T_3_ (3.16 ± 0.01). The lowest fiber content was observed in T_c_ (0.41 ± 0.01) with 5% PPP, while the highest value was found in T_3_ (3.16 ± 0.01) with 15% PPP. The observed data demonstrates a direct relationship between the amount of PPP and the content of dietary fiber, suggesting that PPP can be considered a favorable source of dietary fiber. Aqilah et al. (2023) found increased fiber content in noodles enriched with PPP, aligning with our study's observations. Tharshini (2018) reported higher fiber content in cakes prepared with wheat flour, soybean flour, and PPP compared to the control cake, indicating that fiber content increased with higher PPP amounts. Thus, our investigation on value‐added chapatti aligns with previous studies in terms of fiber content trends. The consumption of PPP chapatti, due to its high fiber content, has the potential to positively impact human health by reducing cholesterol levels, regulating blood sugar levels and blood pressure, as well as preventing constipation and the development of colon cancer (Sharma, 2022).

### 3.2 Sensory analysis

Consumers are more attracted to any food due to its fresh color, high nutritional content, intensified hue, and high caloric value. The color, taste, texture, flavor, and overall acceptability are the main attributes determined in the organoleptic analysis of any product. A 9 hedonic scale method was used to evaluate the organoleptic assays of value‐added chapatti. A panel of jury evaluated the following attributes of products using the 9 hedonic scale method.

#### 3.2.1 Color

Color is a crucial factor in food perception and selection, as it is the first characteristic our senses perceive. It influences acceptability and appetite stimulation. Meal presentation, including color, affects our visual perception and desire for food. Table 3 presents the color results of value‐added chapatti treatments, showing variations among the treatments. T_2_ exhibited the most attractive color, while T_c_ had the highest value (7.3 ± 0.10) and T_3_ had the lowest value (6.8 ± 0.010). Srivastava et al. (2014) found that incorporating dried PPP in multigrain biscuits led to acceptable sensory evaluation, but the color was not appreciated with increased PPP concentration. This trend aligns with the color results of value‐added chapatti. Ismail et al. (2014) also observed a similar trend in the color of cookies when supplemented with pomegranate peel, where increased PPP concentration was not appreciated. Similarly, Alazb et al. (2021) observed a similar trend in the color of muffin cakes made with PPP.

**TABLE 3:** Mean value of sensory evaluation of all treatment chapattis.

	Color	Texture	Flavor	Taste	Overall acceptability
T_C_	7.3 ± 0.10	6.1 ± 0.20	7.1 ± 0.00	7.0 ± 0.20	6.88 ± 0.13
T_1_	7.1 ± 0.00	6.2 ± 0.10	6.5 ± 0.10	7.5 ± 0.00	6.82 ± 0.05
T_2_	7.2 ± 0.20	6.2 ± 0.20	6.8 ± 0.10	7.5 ± 0.10	6.93 ± 0.15
T_3_	6.8 ± .010	6.1 ± 0.20	6.0 ± 0.25	6.5 ± 0.10	6.35 ± 0.17

#### 3.2.2 Flavor

Flavor is a crucial attribute of food, involving the integration of senses such as taste, smell, and tactile sensations in the mouth. Multigrain‐based PPP chapatti exhibited significant flavor variations across different treatments. A panel of judges evaluated the flavor using a 9‐point hedonic scale, and the scores are presented in Table 3. The results showed that treatment T_2_ (6.8 ± 0.10) received the highest score, indicating favorable flavor, while treatment T_3_ (6.0 ± 0.25) had the lowest score. These findings suggest that the flavor of the T_2_ treatment was well appreciated and considered effective. Paul and Bhattacharyya (2015) investigated the sensory properties of cookies fortified with PPP and observed significant changes in flavor with increased powder concentration, which aligns with the results of the current study. Similarly, Topkaya and Isik (2019) developed muffin cakes using PPP and reported that higher amounts of the powder significantly reduced the smell, flavor, and color scores of the products. Therefore, the flavor of the value‐added chapatti corresponds to the findings of previous investigations.

#### 3.2.3 Taste

Taste is a critical attribute in sensory evaluation that directly influences consumer preferences. Consumers are particularly conscious of the taste of food products. Therefore, the taste of the value‐added chapatti was assessed by a panel of judges using a 9‐point hedonic scale. The taste data from different treatments are presented in Table 3. T_c_ had a taste score of (7.0 ± 0.20), while treatments T_1_, T_2_, and T_3_, with 5%, 10%, and 15% PPP, respectively, had scores of (7.5 ± 0.00), (7.5 ± 0.10), and (6.5 ± 0.10). The highest taste score was observed in T_2_ (7.5 ± 0.10), while the lowest score was in T_3_ (6.5 ± 0.10). Statistical analysis confirmed a significant association (*p* < .05). Therefore, based on these results, the T_2_ treatment was selected for further efficacy studies. Aqilah et al. (2023) prepared wheat flour noodles supplemented with dehulled cowpea flour and PPP. They observed that the taste of the noodles decreased with increasing amounts of PPP, which aligns with the taste trend observed in the value‐added chapatti. Additionally, Topkaya and Isik (2019) produced muffin cakes using PPP, finding that higher concentrations of PPP significantly reduced the smell, taste, flavor, and color scores of the products. These findings support the taste results of the value‐added chapatti.

#### 3.2.4 Texture

Texture is an important attribute that encompasses physical characteristics such as shape, tactile sensation, and consistency. When a food item encounters the skin, it stimulates touch receptors and triggers the secretion of mouth fluids. Therefore, texture is a physical parameter of a product that directly interacts with the senses. It provides information about the physical appearance, hardness, and brittleness of the product. The texture results from different treatments are presented in Table 3. The results are as follows: T_c_ (6.1 ± 0.20), T_1_ (6.2 ± 0.10), T_2_ (6.2 ± 0.20), and T_3_ (6.1 ± 0.20). T_2_ (6.2 ± 0.20) exhibited the highest texture score, while T_3_ (6.1 ± 0.20) had the lowest texture score. Statistical analysis revealed a significant association (*p* < .05). Paul and Bhattacharyya (2015) investigated the sensory properties of cookies fortified with PPP and observed a significant change in the physical appearance of cookies, particularly in texture, with an increased amount of PPP. Therefore, the texture of the multigrain chapatti based on PPP also decreased with an increased amount of PPP, which aligns with the findings of the previous study. Alazb et al. (2021) evaluated the sensory characteristics of wheat flour spaghetti supplemented with PPP, and their results are consistent with the texture‐related findings of the current study.

#### 3.2.5 Overall acceptability

**FIGURE 3:**
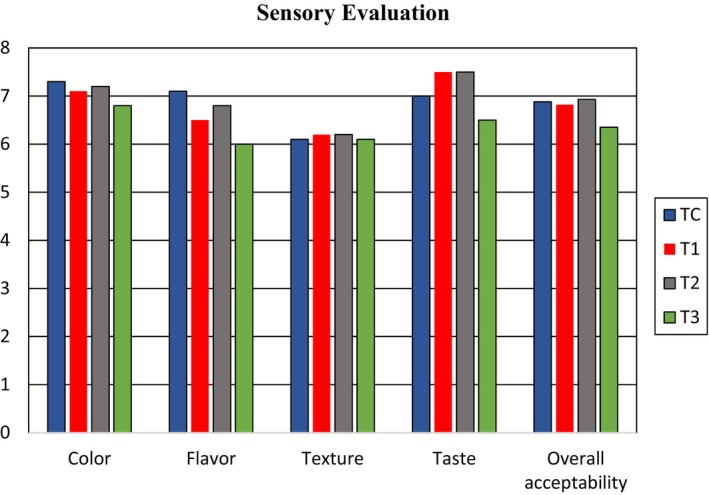
Mean sensory evaluation scores for all treatments.

The overall acceptability of a value‐added product is a crucial organoleptic attribute. The results of the overall acceptability evaluation are presented in Table 3. T_c_ obtained an acceptability score of (6.88 ± 0.13), while treatments T_1_, T_2_, and T_3_, with 5%, 10%, and 15% PPP, respectively, received scores of (6.82 ± 0.05), (6.93 ± 0.15), and (6.35 ± 0.17). T3 (6.35 ± 0.17) had the lowest score, whereas T_2_ (6.93 ± 0.15) exhibited the highest score. Based on these results, it was concluded that T_2_ (6.93 ± 0.15) had excellent overall acceptability compared to the other treatments and was selected for further efficacy studies. Statistical analysis indicated a significant shift in acceptability from T_c_ to T_3_ for the value‐added chapatti. Alazb et al. (2021) investigated the sensory characteristics of wheat flour spaghetti supplemented with different concentrations of PPP (3%, 5%, and 7%). Their sensory evaluation results revealed that substituting wheat flour in spaghetti with up to 7% PPP resulted in satisfactory consumer acceptability. Thus, an increased amount of PPP led to a decreased overall acceptability trend, which is consistent with the findings of the current study. Similarly, Srivastava et al. (2014) assessed the sensory attributes of multigrain biscuits supplemented with PPP at various levels. Their results also showed a decreasing trend in overall acceptability with an increased amount of PPP. Therefore, the current study's findings align with previous research in this regard. A graphical presentation of sensory evaluation is shown in Figure 3.

### 3.3 Physiochemical

#### 3.3.1 Polyphenol content

Polyphenols are a group of plant components considered to enhance digestion and brain function, as well as provide protection against heart disease, type 2 diabetes, and some types of cancer (Rana et al., 2022). The polyphenol content of the selected product (T_2_) was determined using a suitable procedure. The mean values of the polyphenol content are presented in Table 4. The control product (T_c_) had a polyphenol concentration of 17.07 ± 0.31 mg GAE/100 g, whereas the selected product T_2_ had a concentration of 102.11 ± 0.82 mg GAE/100 g. These results indicate that the polyphenol concentration increased with the addition of PPP. Therefore, it can be concluded that incorporating multigrain with PPP enhanced the polyphenol concentration in the value‐added product. This attribute further supports the efficacy of the value‐added chapatti in demonstrating significant results in the study design. Dahiya (2020) investigated the nutritional composition of chapatti prepared with composite flour and found that the polyphenol concentration in composite flour‐based products was higher than that in whole wheat flour. This observation aligns with the current study's findings regarding polyphenols in the value‐added chapatti, providing support for the present scenario. Similarly, Ismail et al. (2014) examined the nutritional composition of PPP‐enriched products and observed that the polyphenol concentration increased with higher amounts of PPP. Therefore, the PPP‐based multigrain chapatti also exhibited an increasing trend in polyphenol concentration, consistent with previous research.

**TABLE 4:** Polyphenol and antioxidant activity of the control and selected products.

	Polyphenol (mg GAE/100 g)	Antioxidant activity (%)
T_c_	17.07 ± 0.31	15.91 ± 0.16
T_2_	102.11 ± 0.82	65.32 ± 0.23

#### 3.3.2 Antioxidant activity (DPPH)

**FIGURE 4:**
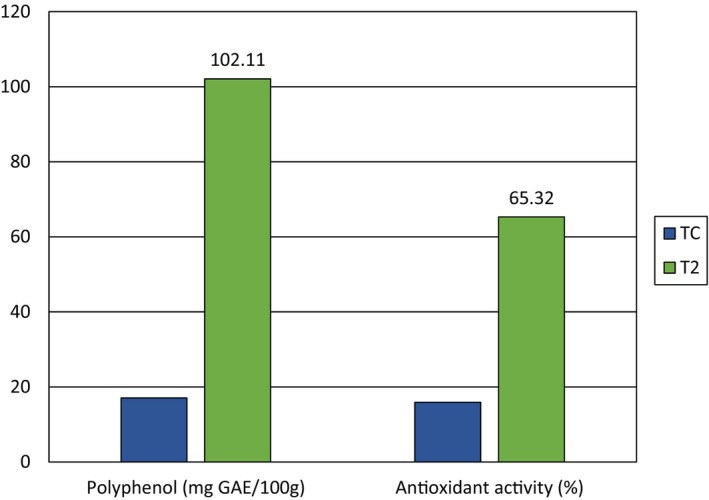
Polyphenol content and antioxidant activity comparison between the control and selected products.

The concentration of DPPH in the PPP‐based chapatti was determined using an appropriate procedure, and the mean values are presented in Table 4. Tc had a DPPH concentration of 15.91 ± 0.16%, while the selected product T_2_ had a concentration of 65.32 ± 0.23%. These results indicate that the DPPH concentration increased with the addition of PPP. Therefore, it can be concluded that incorporating multigrain with PPP enhanced the DPPH concentration in the value‐added product. This attribute further supports the efficacy of the value‐added chapatti in demonstrating significant results in the study design. Ismail et al. (2014) investigated the nutritional composition of PPP‐enriched products and observed that the DPPH concentration increased with higher amounts of PPP. This finding aligns with the current study's results regarding the increasing trend in DPPH concentration in the PPP‐based chapatti, providing further support for the present scenario. Similarly, Dahiya (2020) studied the nutritional composition of chapatti prepared with composite flour and found that the concentration of polyphenols was higher in the composite flour‐based product compared to whole wheat flour. This observation parallels the similar trend observed in the polyphenol concentration of the value‐added chapatti. A higher concentration of DPPH signifies a stronger capacity to provide hydrogen atoms to neutralize free radicals. Antioxidants can eliminate harmful free radicals and mitigate their effects, resulting in several health advantages, including a lower probability of developing chronic illnesses such as cancer, diabetes, and heart disease (Engwa et al., 2022). Antioxidant content is shown in Figure 4.

### 3.4 Anthropometry of participants

#### 3.4.1 Height

Table 5 presents the mean values of height before and after assessment in diabetic patients with and without chapatti treatment. In the diabetic group without chapatti treatment, the mean height before the assessment was 67.00 ± 2.11 inches, and after the assessment, it remained the same at 67.00 ± 2.21 inches. Conversely, in the diabetic group with chapatti treatment, the mean height before the assessment was 66.30 ± 1.77 inches. A statistical analysis was performed to evaluate the association between height and chapatti treatment. The results revealed a high, non‐significant association in the diabetic group without chapatti treatment, suggesting that the consumption of the value‐added product did not have a significant impact on height.

**TABLE 5:** Mean anthropometry values of diabetic patients without chapatti and diabetic patients with chapatti before and after the study.

Characteristics	Diabetic patient without chapatti (*n* = 10)		Diabetic patient with chapatti (*n* = 10)	
	Pre‐assessment	Post‐assessment	Pre‐assessment	Post‐assessment
Height (inches)	67.00 ± 2.11	67.00 ± 2.21	66.30 ± 1.77	66.30 ± 1.77
Weight (kg)	73.65 ± 3.76	73.80 ± 4.64	71.30 ± 3.39	70.20 ± 2.19
BMI (kg/m^2^)	25.41 ± 1.50	25.38 ± 1.71	25.12 ± 1.40	24.73 ± 1.18

#### 3.4.2 Weight

The distribution of individuals within different weight ranges before and after assessment in both the diabetic groups, with and without chapatti treatment, is presented in Table 5. There was no individual observed in the range of <55 kg weight before and after assessment of both diabetic groups without and with chapatti treatment. The mean values of weight before and after assessment in the diabetic group without chapatti treatment are presented in Table 6, where slight variation is exhibited, with values of 73.65 ± 3.76 and 73.80 ± 4.64 kg, respectively. In contrast, the diabetic group with chapatti treatment demonstrated a noticeable decrease in weight during the post‐assessment period (70.20 ± 2.19 kg) compared to the pre‐assessment period (71.30 ± 3.39 kg). A statistical analysis was conducted to evaluate the association between weight and chapatti treatment. The results indicated a non‐significant association in the diabetic group without chapatti treatment (*p* > .05). However, in the diabetic group with chapatti treatment, the statistical analysis revealed a significant association (*p* < .05). These findings suggest that the inclusion of chapatti treatment had a notable impact on weight reduction in diabetic patients.

**TABLE 6:** Mean biochemical assessment values of diabetic patients without and with chapatti treatment before and after the study.

Characteristics	Diabetic patient without chapatti (*n* = 10)		Diabetic patient with chapatti (*n* = 10)	
	Pre‐assessment	Post‐assessment	Pre‐assessment	Post‐assessment
RBG (mg/dL)	239.4 ± 50.6	230.7 ± 47.9	242.4 ± 34.1	219.9 ± 32.6
FBG (mg/dL)	222.0 ± 51.7	212.5 ± 48.62	223.3 ± 33.1	190.9 ± 31.44
HbA1C (%)	8.78 ± 0.78	7.45 ± 0.68	8.52 ± 0.95	6.78 ± 0.52

#### 3.4.3 Body mass index

The distribution of individuals across different BMI ranges before and after assessment in both the diabetic groups, without and with chapatti treatment, is presented in Table 5. It is noteworthy that none of the individuals were classified within the BMI range of less than 18.5 kg/m^2^ before and after assessment in both groups. The mean values of BMI are summarized in Table 6. In the diabetic group without chapatti treatment, the mean BMI before the assessment was calculated as 25.41 ± 1.50 kg/m^2^, and after assessment, it was found to be 25.38 ± 1.71 kg/m^2^. Conversely, in the diabetic group with chapatti treatment, the mean BMI before and after assessment was determined to be 25.12 ± 1.40 and 24.73 ± 1.18 kg/m^2^, respectively. A statistical analysis was performed to assess the association between BMI and chapatti treatment. The results revealed a significant association in the group with chapatti treatment (*p* < .05). These findings suggest that the addition of chapatti treatment had a notable impact on BMI management in diabetic patients. The higher percentage of individuals falling within the BMI range of 18.5 to 24.9 kg/m^2^ after assessment in the group with chapatti treatment indicates a positive effect on maintaining a healthy BMI.

### 3.5 Biochemical assessment

#### 3.5.1 Random blood glucose (RBG)

Random blood glucose is a quantitative assessment of the concentration of glucose in the bloodstream at any given moment. It serves as a reliable method for evaluating the regulation of glucose levels. It is crucial to acknowledge that RBG levels can be impacted by several factors, such as nutrition, physical activity, stress, and the administration of specific drugs (Wronka et al., 2022). Table 6 presents the percentage of individuals in the diabetic groups without and with chapatti treatment, categorized by RBG ranges before and after assessment. The RBG ranges were defined as above 200, 200–250, and above 250 mg/dL. The mean RBG values for the diabetic group without chapatti treatment were 239.4 ± 50.6 mg/dL before assessment and 230.7 ± 47.9 mg/dL after assessment. In contrast, the mean RBG values for the diabetic group with chapatti treatment were 242.4 ± 34.1 mg/dL before assessment and 219.9 ± 32.6 mg/dL after assessment. The statistical analysis of the diabetic group without chapatti treatment showed a non‐significant association (*p* > .05), indicated by a *p*‐value of 0.152 and a *t*‐value of 1.564. However, in the group with chapatti treatment, the statistical analysis revealed a significant association (*p* < .005), with an altered *p*‐value of .004 and a *t*‐value of 3.833. These results suggest that the consumption of value‐added chapatti had a positive effect on reducing RBG concentrations and lowering blood glucose levels. Previous studies by Manna et al. (2019) and Olagunju and Omoba (2022) have also demonstrated significant decreases in blood glucose levels with the use of value‐added products, supporting the findings of our study. These consistent results further highlight the potential benefits of incorporating value‐added components into the diet of diabetic patients for managing RBG levels.

#### 3.5.2 Fasting blood glucose (FBG)

Table 6 displays the percentage of individuals in the diabetic groups without and with chapatti treatment, categorized by fasting blood glucose (FBG) ranges before and after assessment. The FBG ranges included 60–110 mg/dL, 111–249 mg/dL, and ≥250 mg/dL. None of the individuals in either group fell within the normal FBG range (60–110 mg/dL) before and after the assessment. The mean FBG values, as presented in Table 6, for the diabetic group without chapatti treatment were 222.0 ± 51.7 mg/dL before assessment and 212.5 ± 48.62 mg/dL after assessment. In contrast, the mean FBG values for the diabetic group with chapatti treatment were 223.3 ± 33.1 mg/dL before assessment and 190.9 ± 31.44 mg/dL after assessment. The statistical analysis showed a significant association (*p* < .05) between the diabetic individuals in the group with chapatti treatment and FBG concentrations, as evidenced by a lower *p*‐value (.016) and higher *t*‐value (2.941) compared to the group without chapatti treatment. Conversely, the diabetic group without chapatti treatment exhibited a highly non‐significant association (*p* > .05) between FBG levels and assessment. These results indicate that the consumption of value‐added chapatti has a positive effect on lowering FBG concentrations in diabetic patients. The findings of our study align with previous research by Olagunju and Omoba (2022) and Manna et al. (2019), which also reported significant decreases in blood glucose levels with the consumption of multigrain snacks and pomegranate peel, respectively, supporting the consistency of our findings with prior studies.

#### 3.5.3 HbA1C

**FIGURE 5:**
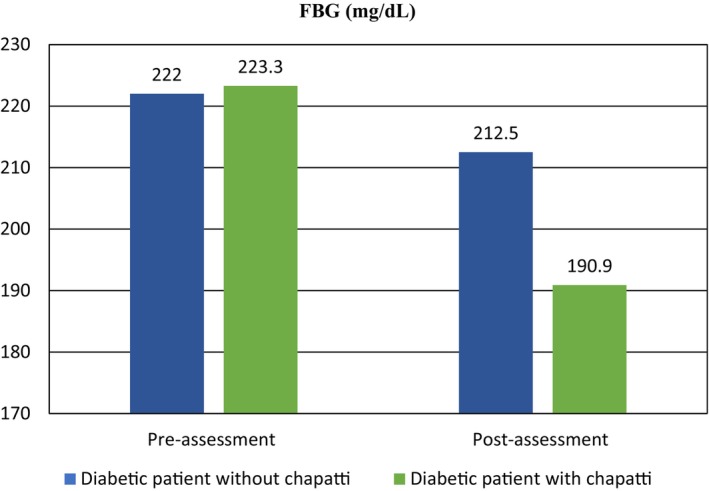
Pre‐ and post‐assessment fasting blood glucose levels comparison in selected groups.

**FIGURE 6:**
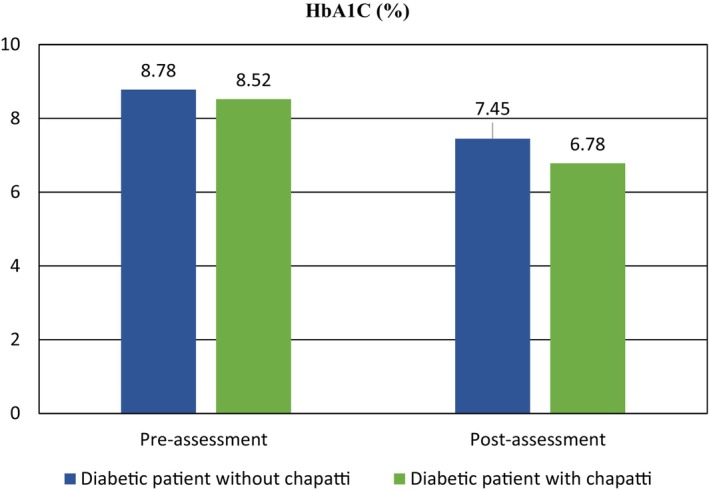
Pre‐ and post‐assessment HbA1c levels comparison in selected groups.

HbA1C is a metric that quantifies the mean blood glucose levels throughout the preceding 2 to 3 months, serving as a reliable indicator of glucose regulation (Al Hayek et al., 2022). Table 6 displays the percentage of individuals in the diabetic groups without and with chapatti treatment, categorized by HbA1C ranges before and after assessment. None of the individuals in either group fell within the normal HbA1C range (4.0–5.7%) before and after the assessment. The mean HbA1C values for the diabetic group without chapatti treatment were 8.78 ± 0.78% before assessment and 7.45 ± 0.68% after assessment. Conversely, the mean HbA1C values for the diabetic group with chapatti treatment were 8.52 ± 0.95% before assessment and 6.78 ± 0.52% after assessment. Notably, the mean HbA1C values were lower in the group with chapatti treatment compared to other groups. The statistical analysis indicated a highly significant association (*p* < .05) between HbA1C levels and both the diabetic group without chapatti treatment and the group with chapatti treatment before and after assessment. These findings suggest that the consumption of multigrain‐based chapatti had a positive impact on reducing HbA1C levels, thereby decreasing the risk of diabetes mellitus. The study by Sardar et al. (2019) demonstrated a significant decrease in HbA1C percentage with exercise and pomegranate consumption in middle‐aged women with metabolic syndrome, supporting the results of our study. Similarly, Yagi et al. (2014) investigated the hypoglycemic effect of pomegranate extract and observed a significant decrease in HbA1C percentage. These findings align with the decrease in HbA1C percentage observed in our study, further emphasizing the beneficial effects of pomegranate consumption. However, all biochemical findings have been mentioned in Figures 5 and 6.

## 4 CONCLUSION

The study highlights the beneficial effects of incorporating PPP into multigrain chapatti, particularly in the T_3_ formulation. The findings underscore the importance of dietary interventions enriched with natural bioactive compounds in promoting metabolic health. Further research is warranted to explore the long‐term effects and mechanisms underlying the observed improvements and to validate the role of PPP‐based multigrain chapatti in diabetes prevention.

## AUTHOR CONTRIBUTIONS

Muhammad Zubair, Aftab Ahmed, Muhammad Afzaal and Farhan Saeed designed the study and conduct under their supervision. Muhammad Zubair, Zargham Faisal and Noor Akram performed the study and participated in drafting the article. Muhammad Afzaal and Zargham Faisal helped in developing the whole concept and editing. Noor Akram and Aasma Asghar helped in preparing Figures and Tables, the overall quality of the manuscript was maintained by Aftab Ahmed. Farhan Saeed, Asad Nawaz and Muhammad Afzaal wrote, edited and revised the manuscript critically. Degnet Teferi Asres and Salim Manoharadas revised the final written paper. The final version of the manuscript has been read and approved by all listed authors.

## FUNDING INFORMATION

The authors declare that no funds, grants, or other support were received during the preparation of this manuscript.

## CONFLICT OF INTEREST STATEMENT

The authors declare no conflict of interest.

## Data Availability

Even though adequate data have been given in the form of tables and figures, all authors declare that if more data are required, then they will be provided on a request basis.
